# {6,6′-Dieth­oxy-2,2′-[2,2-dimethyl­propane-1,3-diylbis(nitrilo­methyl­idyne)]diphenolato}copper(II) monohydrate

**DOI:** 10.1107/S1600536809012859

**Published:** 2009-04-10

**Authors:** Hadi Kargar, Reza Kia, Hoong-Kun Fun, Arezoo Jamshidvand

**Affiliations:** a Department of Chemistry, School of Science, Payame Noor University (PNU), Ardakan, Yazd, Iran; b X-ray Crystallography Unit, School of Physics, Universiti Sains Malaysia, 11800 USM, Penang, Malaysia

## Abstract

In the title complex, [Cu(C_23_H_28_N_2_O_4_)]·H_2_O, the Cu^II^ ion has a distorted planar geometry, coordinated by the N_2_O_2_ unit of the tetra­dentate Schiff base ligand. The asymmetric unit comprises one complex mol­ecule and a water mol­ecule of crystallization. The water H atoms form bifurcated O—H⋯(O,O) inter­molecular hydrogen bonds with the O atoms of the phenolate and eth­oxy groups with *R_1_^2^(5)* and *R_1_^2^(6)* ring motifs, which may, in part, influence the mol­ecular configuration. The dihedral angle between the two O—Cu—N coordination planes is 31.02 (6)° and the dihedral angle between the two benzene rings is 34.98 (7)°. In the crystal structure, mol­ecules are linked together by inter­molecular C—H⋯O inter­actions, forming extended chains along the *a* axis. The crystal structure is further stabilized by inter­molecular C—H⋯π and π–π [centroid–centroid = 3.5068 (13) Å] inter­actions.

## Related literature

For hydrogen-bond motifs, see: Bernstein *et al.* (1995 ▶). For bond-length data, see Allen *et al.* (1987 ▶). For related structures see, for example: Clark *et al.* (1968 ▶, 1969 ▶, 1970 ▶). For applications and bioactivity of Cu(II) and Ni(II) Schiff base complexes see, for example: Elmali *et al.* (2000 ▶); Blower (1998 ▶); Granovski *et al.* (1993 ▶); Li & Chang (1991 ▶); Shahrokhian *et al.* (2000 ▶). For the stability of the temperature controller used for the data collection, see: Cosier & Glazer (1986 ▶).
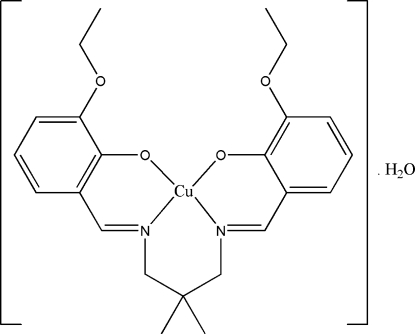


## Experimental

### 

#### Crystal data

[Cu(C_23_H_28_N_2_O_4_)]·H_2_O
*M* *_r_* = 478.03
Triclinic, 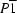

*a* = 9.427 (3) Å
*b* = 10.805 (3) Å
*c* = 12.771 (4) Å
α = 114.554 (13)°
β = 99.479 (14)°
γ = 102.676 (14)°
*V* = 1105.3 (6) Å^3^
*Z* = 2
Mo *K*α radiation
μ = 1.03 mm^−1^
*T* = 100 K
0.50 × 0.22 × 0.15 mm

#### Data collection

Bruker SMART APEXII CCD area-detector diffractometer
Absorption correction: multi-scan (**SADABS**; Bruker, 2005 ▶) *T*_min_ = 0.628, *T*_max_ = 0.861
36656 measured reflections
7929 independent reflections
6977 reflections with *I* > 2σ*I*)
*R*_int_ = 0.029

#### Refinement

*R*[*F* ^2^ > 2σ(*F* ^2^)] = 0.029
*wR*(*F* ^2^) = 0.077
*S* = 1.05
7929 reflections
290 parameters
H atoms treated by a mixture of independent and constrained refinement
Δρ_max_ = 0.58 e Å^−3^
Δρ_min_ = −0.30 e Å^−3^

### 

Data collection: *APEX2* (Bruker, 2005 ▶); cell refinement: *SAINT* (Bruker, 2005 ▶); data reduction: *SAINT*; program(s) used to solve structure: *SHELXTL* (Sheldrick, 2008 ▶); program(s) used to refine structure: *SHELXTL*; molecular graphics: *SHELXTL*; software used to prepare material for publication: *SHELXTL* and *PLATON* (Spek, 2009 ▶).

## Supplementary Material

Crystal structure: contains datablocks global, I. DOI: 10.1107/S1600536809012859/kj2122sup1.cif

Structure factors: contains datablocks I. DOI: 10.1107/S1600536809012859/kj2122Isup2.hkl

Additional supplementary materials:  crystallographic information; 3D view; checkCIF report

## Figures and Tables

**Table 1:** Hydrogen-bond geometry (Å, °)

*D*—H⋯*A*	*D*—H	H⋯*A*	*D*⋯*A*	*D*—H⋯*A*
O1*W*—H2*W*1⋯O2^i^	0.78 (2)	2.41 (2)	2.9959 (18)	132.8 (18)
O1*W*—H2*W*1⋯O4^i^	0.78 (2)	2.27 (2)	3.0097 (19)	159 (2)
O1*W*—H1*W*1⋯O1^i^	0.75 (2)	2.20 (2)	2.8749 (16)	151 (2)
O1*W*—H1*W*1⋯O3^i^	0.75 (2)	2.54 (2)	3.1684 (19)	143 (2)
C7—H7*A*⋯O1*W*	0.95	2.56	3.451 (2)	157
C10—H10*B*⋯O2^ii^	0.99	2.57	3.476 (2)	151
C8—H8*B*⋯*Cg*1^i^	0.99	2.78	3.4918 (19)	129
C13—H13*A*⋯*Cg*1^ii^	0.95	2.85	3.3718 (18)	116
C18—H18*B*⋯*Cg*2^iii^	0.99	2.79	3.718 (2)	157

Symmetry codes: (i) 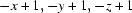; (ii) 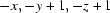; (iii) 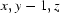. *Cg*1 and *Cg*2 are the centroids of the C1–C6 and C12–C17 benzene rings.

**Fig. 1.**
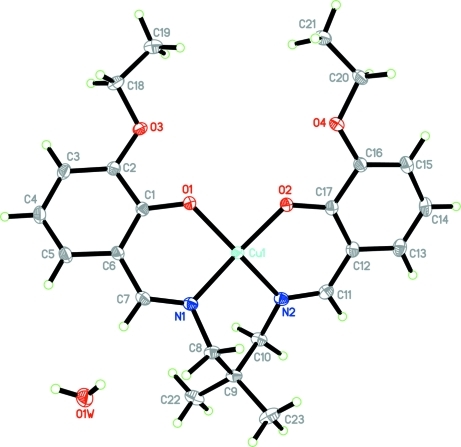
The molecular structure of the title compound, showing 50% probability displacement ellipsoids and the atomic numbering.

**Fig. 2.**
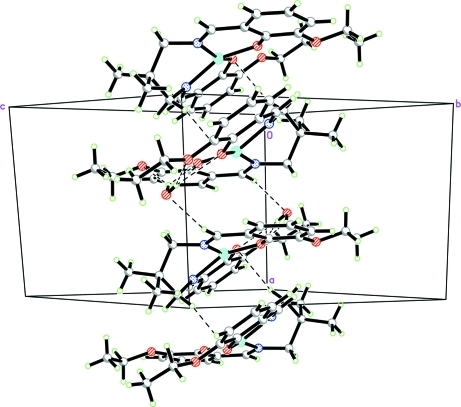
The crystal packing of the title compound, showing 1-D extended chains along the a-axis. Intermolecular interactions are drawn as dashed lines.

## supplementary crystallographic information

### Comment

Schiff base complexes are some of the most important stereochemical models in transition metal coordination chemistry, with their ease of preparation and structural variations (Granovski *et al.*, 1993). Metal derivatives of Schiff bases have been studied extensively, and copper(II) and Ni(II) complexes play a major role in both synthetic and structural research (Elmali *et al.*, 2000; Blower, 1998; Granovski *et al.*, 1993; Li & Chang, 1991; Shahrokhian *et al.*, 2000). Tetradentate Schiff base metal complexes may form *trans* or *cis* planar or tetrahedral structures (Elmali *et al.*, 2000).

The Cu^II^ ion of the title compound (Fig. 1), shows a distorted planar geometry which is coordinated by two imine N atoms and two phenol O atoms of the tetradentate Schiff base ligand. The bond lengths (Allen *et al.,*, 1987) and angles are within normal ranges and are comparable with the related structures (Clark *et al.,* 1968, 1969, 1970). The asymmetric unit of the title compound comprises one molecule of complex and a water molecule of crystallization. The water H atoms form bifurcated O—H···(O,O) intermolecular hydrogen bonds with the O atoms of the phenolato and ethoxy groups with *R_1_^2^(5)* and *R_1_^2^(6)* ring motifs (Bernstein *et al.,* 1995), which may, in part, influence the molecular configuration. The dihedral angle between the two benzene rings is 34.98 (7)°. In the crystal structure, the molecules are linked together by intermolecular C—H···O interactions, forming 1-D extended chains along the *a* axis (Fig. 2). The crystal structure is further stabilized by intermolecular C—H···π (Table 1) and π–π interactions [*Cg*3···*Cg*3^i^ = 3.5068 (13) Å, *Cg*3 is the centroid of the Cu1/O1/C1/C6/C7/N1 ring, symmetry operation i = 1-x, 1-y, 1-z].

### Experimental

A chloroform solution (40 ml) of [*N,N'*-Bis(3-ethoxy-salicylidene)-2, 2-dimethyl-1,3-propanediamin (1 mmol, 399 mg) was added to an ethanol solution (20 ml) of CuCl_2_.4H_2_O (1.05 mmol, 216 mg). The mixture was refluxed for 30 min and then filtered. After keeping the filtrate in air, green plate-shaped crystals were formed at the bottom of the vessel on slow evaporation of the solvent.

### Refinement

The water H-atoms were located from the difference Fourier map and freely refined. The rest of the hydrogen atoms were positioned geometrically [C—H = 0.95–99 Å] and refined using a riding approximation model with U_iso_(H) = 1.2 or 1.5 U_eq_(C). A rotating-group model was used for the methyl groups of the ethoxy substituents.

### Crystal data

**Table:**

[Cu(C_23_H_28_N_2_O_4_)]·H_2_O	*Z* = 2
*M**_r_* = 478.03	*F*(000) = 502
Triclinic, *P*1	*D*_x_ = 1.436 Mg m^−^^3^
Hall symbol: -P 1	Mo *K*α radiation, λ = 0.71073 Å
*a* = 9.427 (3) Å	Cell parameters from 9641 reflections
*b* = 10.805 (3) Å	θ = 2.5–36.5°
*c* = 12.771 (4) Å	µ = 1.02 mm^−^^1^
α = 114.554 (13)°	*T* = 100 K
β = 99.479 (14)°	Plate, green
γ = 102.676 (14)°	0.50 × 0.22 × 0.15 mm
*V* = 1105.3 (6) Å^3^	

### Data collection

**Table:**

Bruker SMART APEXII CCD area-detector diffractometer	7929 independent reflections
Radiation source: fine-focus sealed tube	6977 reflections with *I* > 2˘*I*)
graphite	*R*_int_ = 0.029
φ and ω scans	θ_max_ = 32.5°, θ_min_ = 2.1°
Absorption correction: multi-scan (*SADABS*; Bruker, 2005)	*h* = −14→13
*T*_min_ = 0.628, *T*_max_ = 0.861	*k* = −16→16
36656 measured reflections	*l* = −19→18

### Refinement

**Table:**

Refinement on *F*^2^	Primary atom site location: structure-invariant direct methods
Least-squares matrix: full	Secondary atom site location: difference Fourier map
*R*[*F*^2^ > 2σ(*F*^2^)] = 0.029	Hydrogen site location: inferred from neighbouring sites
*wR*(*F*^2^) = 0.077	H atoms treated by a mixture of independent and constrained refinement
*S* = 1.05	*w* = 1/[σ^2^(*F*_o_^2^) + (0.035*P*)^2^ + 0.4488*P*] where *P* = (*F*_o_^2^ + 2*F*_c_^2^)/3
7929 reflections	(Δ/σ)_max_ = 0.001
290 parameters	Δρ_max_ = 0.58 e Å^−^^3^
0 restraints	Δρ_min_ = −0.30 e Å^−^^3^

### Special details

**Table:**

Experimental. The crystal was placed in the cold stream of an Oxford Cyrosystems Cobra open-flow nitrogen cryostat (Cosier & Glazer, 1986) operating at 100.0 (1)K.
Geometry. All esds (except the esd in the dihedral angle between two l.s. planes) are estimated using the full covariance matrix. The cell esds are taken into account individually in the estimation of esds in distances, angles and torsion angles; correlations between esds in cell parameters are only used when they are defined by crystal symmetry. An approximate (isotropic) treatment of cell esds is used for estimating esds involving l.s. planes.
Refinement. Refinement of F^2^ against ALL reflections. The weighted R-factor wR and goodness of fit S are based on F^2^, conventional R-factors R are based on F, with F set to zero for negative F^2^. The threshold expression of F^2^ > 2sigma(F^2^) is used only for calculating R-factors(gt) etc. and is not relevant to the choice of reflections for refinement. R-factors based on F^2^ are statistically about twice as large as those based on F, and R- factors based on ALL data will be even larger.

### Fractional atomic coordinates and isotropic or equivalent isotropic displacement parameters (Å^2^)

**Table:**

	*x*	*y*	*z*	*U*_iso_*/*U*_eq_	
Cu1	0.234895 (16)	0.580803 (14)	0.526101 (11)	0.01366 (4)	
O1	0.29055 (10)	0.46744 (9)	0.59658 (7)	0.01601 (15)	
O2	0.25360 (10)	0.72661 (9)	0.68076 (7)	0.01635 (16)	
O3	0.30299 (10)	0.32190 (9)	0.71356 (7)	0.01808 (16)	
O4	0.28979 (11)	0.88036 (9)	0.90698 (7)	0.01849 (16)	
N1	0.28983 (11)	0.47831 (10)	0.38154 (8)	0.01475 (17)	
N2	0.09621 (11)	0.64285 (10)	0.44256 (8)	0.01524 (17)	
C1	0.32220 (13)	0.34876 (12)	0.54318 (10)	0.01426 (19)	
C2	0.33163 (13)	0.26509 (12)	0.60485 (10)	0.01502 (19)	
C3	0.36453 (14)	0.13847 (13)	0.55507 (11)	0.0185 (2)	
H3A	0.3662	0.0827	0.5961	0.022*	
C4	0.39567 (15)	0.09205 (13)	0.44348 (11)	0.0213 (2)	
H4A	0.4200	0.0056	0.4097	0.026*	
C5	0.39097 (15)	0.17149 (13)	0.38339 (10)	0.0195 (2)	
H5A	0.4155	0.1411	0.3094	0.023*	
C6	0.35016 (14)	0.29772 (12)	0.42996 (10)	0.0157 (2)	
C7	0.34006 (13)	0.37055 (12)	0.35797 (10)	0.0161 (2)	
H7A	0.3729	0.3366	0.2877	0.019*	
C8	0.29016 (14)	0.54430 (13)	0.30186 (10)	0.0167 (2)	
H8A	0.3433	0.5009	0.2424	0.020*	
H8B	0.3475	0.6481	0.3501	0.020*	
C9	0.12840 (14)	0.52549 (13)	0.23410 (10)	0.0162 (2)	
C10	0.02042 (13)	0.54321 (13)	0.31412 (10)	0.0168 (2)	
H10A	−0.0582	0.5781	0.2848	0.020*	
H10B	−0.0317	0.4478	0.3054	0.020*	
C11	0.04829 (13)	0.75010 (12)	0.49386 (10)	0.0165 (2)	
H11A	−0.0189	0.7694	0.4429	0.020*	
C12	0.08778 (13)	0.84286 (12)	0.62132 (10)	0.0160 (2)	
C13	0.01217 (14)	0.94670 (13)	0.65979 (11)	0.0197 (2)	
H13A	−0.0506	0.9603	0.6019	0.024*	
C14	0.02888 (15)	1.02735 (13)	0.77935 (12)	0.0218 (2)	
H14A	−0.0230	1.0958	0.8041	0.026*	
C15	0.12292 (15)	1.00898 (13)	0.86580 (11)	0.0198 (2)	
H15A	0.1347	1.0657	0.9488	0.024*	
C16	0.19833 (14)	0.90889 (12)	0.83082 (10)	0.0160 (2)	
C17	0.18268 (13)	0.82185 (12)	0.70661 (10)	0.01464 (19)	
C18	0.35853 (15)	0.27727 (14)	0.79952 (11)	0.0194 (2)	
H18A	0.4697	0.2970	0.8158	0.023*	
H18B	0.3091	0.1731	0.7685	0.023*	
C19	0.32030 (16)	0.36231 (15)	0.91229 (11)	0.0232 (2)	
H19A	0.3624	0.3404	0.9756	0.035*	
H19B	0.2097	0.3367	0.8961	0.035*	
H19C	0.3639	0.4652	0.9386	0.035*	
C20	0.29694 (16)	0.94726 (13)	1.03175 (10)	0.0213 (2)	
H20A	0.1935	0.9300	1.0414	0.026*	
H20B	0.3479	1.0522	1.0691	0.026*	
C21	0.38663 (17)	0.88079 (14)	1.08999 (11)	0.0240 (3)	
H21A	0.3850	0.9158	1.1738	0.036*	
H21B	0.4918	0.9075	1.0875	0.036*	
H21C	0.3412	0.7760	1.0465	0.036*	
C22	0.05721 (16)	0.37457 (14)	0.12689 (11)	0.0237 (2)	
H22A	−0.0450	0.3641	0.0848	0.036*	
H22B	0.0510	0.3029	0.1556	0.036*	
H22C	0.1201	0.3602	0.0716	0.036*	
C23	0.14514 (17)	0.63837 (16)	0.19007 (13)	0.0265 (3)	
H23A	0.0448	0.6304	0.1467	0.040*	
H23B	0.2098	0.6225	0.1362	0.040*	
H23C	0.1916	0.7345	0.2591	0.040*	
O1W	0.54758 (13)	0.33956 (12)	0.15477 (9)	0.0257 (2)	
H2W1	0.592 (2)	0.287 (2)	0.1566 (18)	0.038 (5)*	
H1W1	0.595 (2)	0.409 (2)	0.2094 (19)	0.037 (5)*	

### Atomic displacement parameters (Å^2^)

**Table:**

	*U*^11^	*U*^22^	*U*^33^	*U*^12^	*U*^13^	*U*^23^
Cu1	0.01571 (7)	0.01562 (7)	0.01206 (6)	0.00751 (5)	0.00543 (5)	0.00692 (5)
O1	0.0211 (4)	0.0163 (4)	0.0143 (3)	0.0101 (3)	0.0072 (3)	0.0077 (3)
O2	0.0201 (4)	0.0175 (4)	0.0143 (3)	0.0106 (3)	0.0065 (3)	0.0073 (3)
O3	0.0224 (4)	0.0241 (4)	0.0159 (4)	0.0136 (3)	0.0081 (3)	0.0128 (3)
O4	0.0248 (5)	0.0187 (4)	0.0132 (3)	0.0098 (3)	0.0070 (3)	0.0067 (3)
N1	0.0145 (4)	0.0175 (4)	0.0133 (4)	0.0055 (3)	0.0050 (3)	0.0075 (3)
N2	0.0138 (4)	0.0179 (4)	0.0141 (4)	0.0050 (4)	0.0044 (3)	0.0076 (3)
C1	0.0125 (5)	0.0153 (4)	0.0145 (4)	0.0054 (4)	0.0039 (4)	0.0061 (4)
C2	0.0125 (5)	0.0181 (5)	0.0153 (4)	0.0064 (4)	0.0040 (4)	0.0079 (4)
C3	0.0184 (6)	0.0188 (5)	0.0207 (5)	0.0095 (4)	0.0045 (4)	0.0101 (4)
C4	0.0236 (6)	0.0190 (5)	0.0216 (5)	0.0129 (5)	0.0061 (5)	0.0070 (4)
C5	0.0212 (6)	0.0207 (5)	0.0168 (5)	0.0117 (5)	0.0069 (4)	0.0059 (4)
C6	0.0171 (5)	0.0174 (5)	0.0135 (4)	0.0090 (4)	0.0052 (4)	0.0062 (4)
C7	0.0157 (5)	0.0187 (5)	0.0133 (4)	0.0061 (4)	0.0057 (4)	0.0061 (4)
C8	0.0160 (5)	0.0212 (5)	0.0157 (5)	0.0055 (4)	0.0063 (4)	0.0108 (4)
C9	0.0155 (5)	0.0207 (5)	0.0146 (4)	0.0050 (4)	0.0053 (4)	0.0102 (4)
C10	0.0127 (5)	0.0210 (5)	0.0142 (4)	0.0036 (4)	0.0032 (4)	0.0072 (4)
C11	0.0130 (5)	0.0188 (5)	0.0190 (5)	0.0051 (4)	0.0035 (4)	0.0104 (4)
C12	0.0136 (5)	0.0153 (5)	0.0188 (5)	0.0050 (4)	0.0042 (4)	0.0076 (4)
C13	0.0162 (5)	0.0172 (5)	0.0245 (5)	0.0070 (4)	0.0033 (4)	0.0087 (4)
C14	0.0204 (6)	0.0164 (5)	0.0273 (6)	0.0094 (4)	0.0081 (5)	0.0069 (4)
C15	0.0217 (6)	0.0159 (5)	0.0204 (5)	0.0070 (4)	0.0089 (4)	0.0057 (4)
C16	0.0173 (5)	0.0150 (5)	0.0161 (5)	0.0056 (4)	0.0062 (4)	0.0070 (4)
C17	0.0143 (5)	0.0134 (4)	0.0170 (5)	0.0042 (4)	0.0060 (4)	0.0073 (4)
C18	0.0182 (6)	0.0266 (6)	0.0212 (5)	0.0099 (5)	0.0064 (4)	0.0168 (5)
C19	0.0242 (6)	0.0287 (6)	0.0191 (5)	0.0075 (5)	0.0059 (5)	0.0140 (5)
C20	0.0323 (7)	0.0171 (5)	0.0136 (5)	0.0082 (5)	0.0093 (5)	0.0052 (4)
C21	0.0349 (7)	0.0206 (5)	0.0152 (5)	0.0074 (5)	0.0066 (5)	0.0082 (4)
C22	0.0228 (6)	0.0276 (6)	0.0151 (5)	0.0043 (5)	0.0051 (4)	0.0068 (4)
C23	0.0235 (6)	0.0352 (7)	0.0322 (7)	0.0095 (6)	0.0085 (5)	0.0259 (6)
O1W	0.0312 (6)	0.0263 (5)	0.0172 (4)	0.0159 (4)	0.0028 (4)	0.0062 (4)

### Geometric parameters (Å, °)

**Table:**

Cu1—O2	1.8952 (10)	C10—H10B	0.9900
Cu1—O1	1.9049 (9)	C11—C12	1.4400 (16)
Cu1—N1	1.9417 (11)	C11—H11A	0.9500
Cu1—N2	1.9536 (11)	C12—C17	1.4185 (16)
O1—C1	1.3064 (13)	C12—C13	1.4200 (16)
O2—C17	1.3066 (13)	C13—C14	1.3675 (18)
O3—C2	1.3656 (14)	C13—H13A	0.9500
O3—C18	1.4398 (14)	C14—C15	1.4076 (18)
O4—C16	1.3689 (14)	C14—H14A	0.9500
O4—C20	1.4326 (14)	C15—C16	1.3838 (16)
N1—C7	1.2915 (15)	C15—H15A	0.9500
N1—C8	1.4653 (15)	C16—C17	1.4299 (16)
N2—C11	1.2933 (15)	C18—C19	1.5049 (18)
N2—C10	1.4728 (15)	C18—H18A	0.9900
C1—C6	1.4109 (15)	C18—H18B	0.9900
C1—C2	1.4317 (15)	C19—H19A	0.9800
C2—C3	1.3786 (16)	C19—H19B	0.9800
C3—C4	1.4061 (17)	C19—H19C	0.9800
C3—H3A	0.9500	C20—C21	1.5123 (19)
C4—C5	1.3713 (18)	C20—H20A	0.9900
C4—H4A	0.9500	C20—H20B	0.9900
C5—C6	1.4120 (16)	C21—H21A	0.9800
C5—H5A	0.9500	C21—H21B	0.9800
C6—C7	1.4418 (16)	C21—H21C	0.9800
C7—H7A	0.9500	C22—H22A	0.9800
C8—C9	1.5487 (17)	C22—H22B	0.9800
C8—H8A	0.9900	C22—H22C	0.9800
C8—H8B	0.9900	C23—H23A	0.9800
C9—C23	1.5308 (17)	C23—H23B	0.9800
C9—C22	1.5315 (18)	C23—H23C	0.9800
C9—C10	1.5436 (16)	O1W—H2W1	0.78 (2)
C10—H10A	0.9900	O1W—H1W1	0.75 (2)
			
O2—Cu1—O1	89.59 (4)	C12—C11—H11A	117.1
O2—Cu1—N1	158.91 (4)	C17—C12—C13	120.39 (11)
O1—Cu1—N1	93.25 (4)	C17—C12—C11	122.37 (10)
O2—Cu1—N2	93.89 (4)	C13—C12—C11	116.79 (10)
O1—Cu1—N2	156.16 (4)	C14—C13—C12	120.65 (11)
N1—Cu1—N2	91.92 (5)	C14—C13—H13A	119.7
C1—O1—Cu1	126.73 (7)	C12—C13—H13A	119.7
C17—O2—Cu1	127.10 (8)	C13—C14—C15	120.06 (11)
C2—O3—C18	117.73 (9)	C13—C14—H14A	120.0
C16—O4—C20	118.24 (9)	C15—C14—H14A	120.0
C7—N1—C8	119.06 (10)	C16—C15—C14	120.49 (11)
C7—N1—Cu1	126.05 (8)	C16—C15—H15A	119.8
C8—N1—Cu1	114.43 (8)	C14—C15—H15A	119.8
C11—N2—C10	117.74 (10)	O4—C16—C15	125.27 (10)
C11—N2—Cu1	125.32 (8)	O4—C16—C17	113.69 (10)
C10—N2—Cu1	115.96 (8)	C15—C16—C17	121.03 (11)
O1—C1—C6	124.96 (10)	O2—C17—C12	125.30 (10)
O1—C1—C2	117.64 (10)	O2—C17—C16	117.30 (10)
C6—C1—C2	117.40 (10)	C12—C17—C16	117.38 (10)
O3—C2—C3	124.88 (10)	O3—C18—C19	106.52 (10)
O3—C2—C1	113.66 (10)	O3—C18—H18A	110.4
C3—C2—C1	121.45 (10)	C19—C18—H18A	110.4
C2—C3—C4	119.90 (11)	O3—C18—H18B	110.4
C2—C3—H3A	120.0	C19—C18—H18B	110.4
C4—C3—H3A	120.0	H18A—C18—H18B	108.6
C5—C4—C3	120.03 (11)	C18—C19—H19A	109.5
C5—C4—H4A	120.0	C18—C19—H19B	109.5
C3—C4—H4A	120.0	H19A—C19—H19B	109.5
C4—C5—C6	120.94 (11)	C18—C19—H19C	109.5
C4—C5—H5A	119.5	H19A—C19—H19C	109.5
C6—C5—H5A	119.5	H19B—C19—H19C	109.5
C1—C6—C5	120.15 (10)	O4—C20—C21	106.68 (10)
C1—C6—C7	122.48 (10)	O4—C20—H20A	110.4
C5—C6—C7	117.36 (10)	C21—C20—H20A	110.4
N1—C7—C6	125.07 (10)	O4—C20—H20B	110.4
N1—C7—H7A	117.5	C21—C20—H20B	110.4
C6—C7—H7A	117.5	H20A—C20—H20B	108.6
N1—C8—C9	112.96 (10)	C20—C21—H21A	109.5
N1—C8—H8A	109.0	C20—C21—H21B	109.5
C9—C8—H8A	109.0	H21A—C21—H21B	109.5
N1—C8—H8B	109.0	C20—C21—H21C	109.5
C9—C8—H8B	109.0	H21A—C21—H21C	109.5
H8A—C8—H8B	107.8	H21B—C21—H21C	109.5
C23—C9—C22	110.02 (10)	C9—C22—H22A	109.5
C23—C9—C10	110.56 (10)	C9—C22—H22B	109.5
C22—C9—C10	106.70 (10)	H22A—C22—H22B	109.5
C23—C9—C8	106.81 (10)	C9—C22—H22C	109.5
C22—C9—C8	110.29 (10)	H22A—C22—H22C	109.5
C10—C9—C8	112.48 (9)	H22B—C22—H22C	109.5
N2—C10—C9	114.22 (10)	C9—C23—H23A	109.5
N2—C10—H10A	108.7	C9—C23—H23B	109.5
C9—C10—H10A	108.7	H23A—C23—H23B	109.5
N2—C10—H10B	108.7	C9—C23—H23C	109.5
C9—C10—H10B	108.7	H23A—C23—H23C	109.5
H10A—C10—H10B	107.6	H23B—C23—H23C	109.5
N2—C11—C12	125.77 (11)	H2W1—O1W—H1W1	103 (2)
N2—C11—H11A	117.1		
			
O2—Cu1—O1—C1	−170.54 (10)	Cu1—N1—C7—C6	6.52 (17)
N1—Cu1—O1—C1	−11.46 (10)	C1—C6—C7—N1	−7.79 (19)
N2—Cu1—O1—C1	90.74 (13)	C5—C6—C7—N1	172.71 (12)
O1—Cu1—O2—C17	−153.04 (10)	C7—N1—C8—C9	114.46 (12)
N1—Cu1—O2—C17	108.98 (13)	Cu1—N1—C8—C9	−72.85 (11)
N2—Cu1—O2—C17	3.35 (10)	N1—C8—C9—C23	161.31 (10)
O2—Cu1—N1—C7	99.12 (14)	N1—C8—C9—C22	−79.16 (12)
O1—Cu1—N1—C7	1.82 (10)	N1—C8—C9—C10	39.83 (13)
N2—Cu1—N1—C7	−154.90 (10)	C11—N2—C10—C9	123.50 (12)
O2—Cu1—N1—C8	−72.98 (14)	Cu1—N2—C10—C9	−67.24 (11)
O1—Cu1—N1—C8	−170.28 (8)	C23—C9—C10—N2	−88.70 (12)
N2—Cu1—N1—C8	33.01 (8)	C22—C9—C10—N2	151.68 (10)
O2—Cu1—N2—C11	−0.13 (10)	C8—C9—C10—N2	30.61 (14)
O1—Cu1—N2—C11	97.69 (13)	C10—N2—C11—C12	167.99 (11)
N1—Cu1—N2—C11	−159.83 (10)	Cu1—N2—C11—C12	−0.16 (17)
O2—Cu1—N2—C10	−168.46 (8)	N2—C11—C12—C17	−2.29 (19)
O1—Cu1—N2—C10	−70.64 (13)	N2—C11—C12—C13	−174.60 (12)
N1—Cu1—N2—C10	31.83 (8)	C17—C12—C13—C14	−0.38 (19)
Cu1—O1—C1—C6	13.28 (17)	C11—C12—C13—C14	172.08 (12)
Cu1—O1—C1—C2	−167.75 (8)	C12—C13—C14—C15	0.7 (2)
C18—O3—C2—C3	21.84 (17)	C13—C14—C15—C16	−0.4 (2)
C18—O3—C2—C1	−159.14 (10)	C20—O4—C16—C15	6.95 (18)
O1—C1—C2—O3	0.97 (15)	C20—O4—C16—C17	−171.68 (10)
C6—C1—C2—O3	−179.98 (10)	C14—C15—C16—O4	−178.61 (12)
O1—C1—C2—C3	−179.98 (11)	C14—C15—C16—C17	−0.07 (19)
C6—C1—C2—C3	−0.92 (17)	Cu1—O2—C17—C12	−6.51 (17)
O3—C2—C3—C4	−178.50 (11)	Cu1—O2—C17—C16	171.46 (8)
C1—C2—C3—C4	2.56 (19)	C13—C12—C17—O2	177.86 (11)
C2—C3—C4—C5	−1.0 (2)	C11—C12—C17—O2	5.82 (18)
C3—C4—C5—C6	−2.1 (2)	C13—C12—C17—C16	−0.11 (17)
O1—C1—C6—C5	176.78 (11)	C11—C12—C17—C16	−172.14 (11)
C2—C1—C6—C5	−2.20 (17)	O4—C16—C17—O2	0.89 (15)
O1—C1—C6—C7	−2.71 (19)	C15—C16—C17—O2	−177.80 (11)
C2—C1—C6—C7	178.31 (11)	O4—C16—C17—C12	179.03 (10)
C4—C5—C6—C1	3.77 (19)	C15—C16—C17—C12	0.33 (17)
C4—C5—C6—C7	−176.71 (12)	C2—O3—C18—C19	176.50 (10)
C8—N1—C7—C6	178.29 (11)	C16—O4—C20—C21	172.02 (10)

### Hydrogen-bond geometry (Å, °)

**Table:**

*D*—H···*A*	*D*—H	H···*A*	*D*···*A*	*D*—H···*A*
O1W—H2W1···O2^i^	0.78 (2)	2.41 (2)	2.9959 (18)	132.8 (18)
O1W—H2W1···O4^i^	0.78 (2)	2.27 (2)	3.0097 (19)	159 (2)
O1W—H1W1···O1^i^	0.75 (2)	2.20 (2)	2.8749 (16)	151 (2)
O1W—H1W1···O3^i^	0.75 (2)	2.54 (2)	3.1684 (19)	143 (2)
C7—H7A···O1W	0.95	2.56	3.451 (2)	157
C10—H10B···O2^ii^	0.99	2.57	3.476 (2)	151
C8—H8B···Cg1^i^	0.99	2.78	3.4918 (19)	129
C13—H13A···Cg1^ii^	0.95	2.85	3.3718 (18)	116
C18—H18B···Cg2^iii^	0.99	2.79	3.718 (2)	157

Symmetry codes: (i) −*x*+1, −*y*+1, −*z*+1; (ii) −*x*, −*y*+1, −*z*+1; (iii) *x*, *y*−1, *z*.
